# Supplementary material for: The evolution of hematopoietic cells under cancer therapy
Source: Nat Commun. 2021 Aug 10;12:4803. doi: 10.1038/s41467-021-24858-3 (PMC8355079; doi:10.1038/s41467-021-24858-3)

Supplementary Information

The evolution of hematopoietic cells under cancer therapy

Oriol Pich 1 , Albert Cortes-Bullich 2 , Ferran Muiños 1 , Marta Pratcorona 2 , Abel Gonzalez-Perez 1,3,* , Nuria Lopez-Bigas 1,3,4,*

1 Institute for Research in Biomedicine (IRB Barcelona), The Barcelona Institute of Science and Technology, Baldiri Reixac, 10, 08028 Barcelona, Spain.
2 Hematology and Hemotherapy Department, Hospital Santa Creu i Sant Pau, Carrer de Sant Quintí, 89, 08026 Barcelona
3 Research Program on Biomedical Informatics, Universitat Pompeu Fabra, Barcelona, Catalonia, Spain.
4 Institució Catalana de Recerca i Estudis Avançats (ICREA), Barcelona, Spain.
*Corresponding authors: abel.gonzalez@irbbarcelona, nuria.lopez@irbbarcelona.org

Supplementary Figures
Supplementary Tables
Supplementary Notes

Supplementary Figure 1

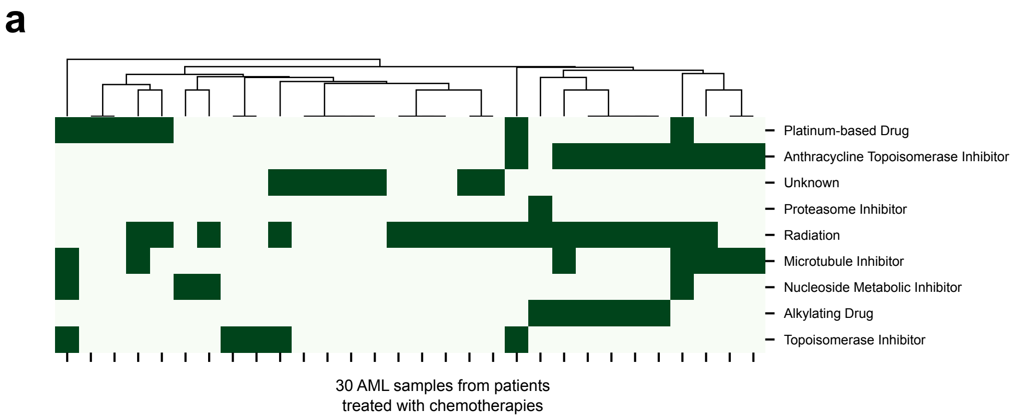

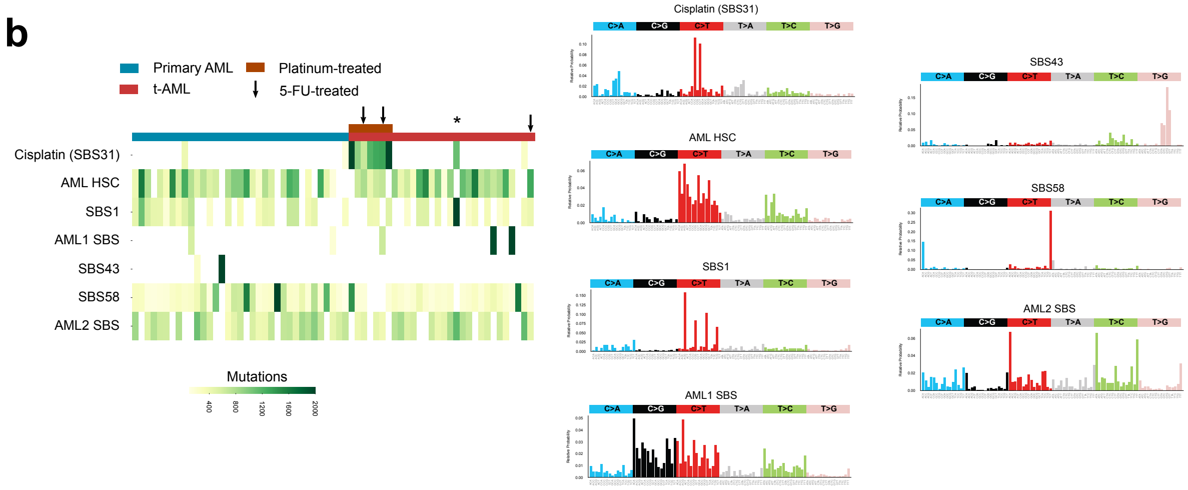

Figure S1. The mutational footprint of chemotherapies across treatment-related AMLs

a) Anticancer treatments to which patients in the WGS AML cohort were exposed as part of the treatment of their primary solid tumors.

b) Mutational signatures active across the WGS AML cohort (right panel), and their activity in each sample of the cohort (left). Mutational signatures with known etiology are referred to by their origin, or their number in the compendium of mutational signatures maintained by the COSMIC repository. A tAML case with activity of the platinum-related signature (and the highest number of double-base substitutions in the entire cohort) from a patient who according to their clinical data was not exposed to any platinum-based drug is marked with an asterisk. We reasoned that, despite the absence of record, a platinum-based drug was part of the treatment of this patient for their primary tumor. One non-platinum-exposed tAML and one primary AML case exhibit a small activity (below 10% of the samples mutation burden) of the platinum-related signature. This is probably the result of "bleeding" and not a real contribution to their mutation profile. These two samples exhibit 30 and 35 DBS, a number closer to the median DBS burden of primary and non-platinum-exposed tAMLs (28) than to that of platinum-exposed tAMLs (48). A mutational signature of unknown etiology (AML1 SBS) is active in two tAML cases, which were exposed to two cytidine analogs employed in the treatment of myelodysplastic syndrome (azacitidine and decitabine). Nevertheless, whether the exposure to these drugs is connected to the etiology of this signature remains to be investigated.

AML, Acute Myeloid Leukemia; t-AML, treatment-related AML; 5-FU, 5-fluorouracil; SBS31, platinum-related single base substitution signature; HSC Hematopoietic Stem Cell; AML HSC, HSC signature; SBS1, age-related single base substitution signature; SBS43, SBS58; single base substitution signatures likely related to sequencing artifacts.

Supplementary Figure 2

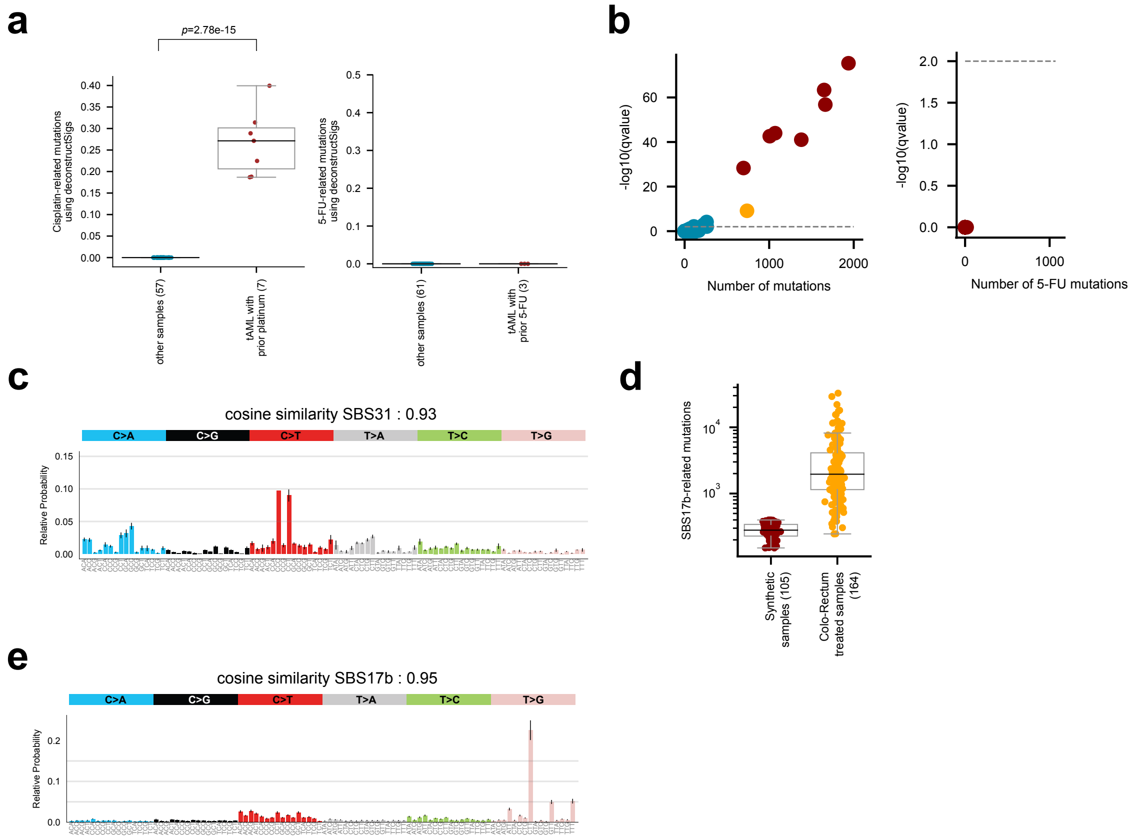

Figure S2. Absence of the 5FU-related signatures from exposed tAML cases

a) Contribution of the platinum-related (left) and the 5FU-related (right) signatures to the reconstruction (using the deconstructSigs algorithm) to the mutational profile of each AML sample in the WGS AML cohort, measured as the estimated percentage of mutations contributed by the signatures. Samples are represented as dots and separated into two groups depending on their known exposure (or absence thereof) to the corresponding chemotherapy. While more than 25% of mutations of every platinum-exposed tAML are contributed by the platinum-related signature, no mutations appear contributed by the 5FU-related signature in none of the 3 5FU-exposed tAMLs. The difference between the distribution of platinum signature activity of both groups of samples is significant (two-tailed Mann-Whitney).

b) Contribution of the platinum-related (left) and the 5FU-related (right) signatures to the reconstruction (using the mSigAct algorithm) of the mutational profile of each AML sample in the WES AML cohort. In this case, the significance of the improvement of the reconstruction when the corresponding signature is included in the analysis, with respect to the background (all other active signatures shown in Fig. S1b), and the estimated number of mutations contributed by the signature to the sample are computed. While the reconstruction of the mutational profile of every platinum-exposed tAML (red dots) improves significantly with the inclusion of the platinum-related signature, those of 5FU-exposed tAMLs do not improve significantly by the inclusion of the 5FU-related signature.

c) Average mutational profile of the platinum-related signature obtained from 35 de novo signatures extraction from all possible random combinations of 3 platinum-exposed tAMLs and all not-platinum-exposed tAMLs in the WGS AML cohort. In the 35 de novo extractions the platinum-related signature (i.e., with cosine similarity to SBS31 above 0.9). Overall, the 35 platinum-related signatures extracted exhibited a mean cosine similarity of 0.936 with SBS31.

d) Thirty-five groups of 3 synthetic samples with the same burden and mutational profile of AMLs were created and a variable number of mutations following the 5FU tri-nucleotide probabilities were injected in each of them. The plot shows the distribution of the number of 5FU-related mutations injected across the 105 synthetic samples (left) and observed across colorectal metastatic tumors from patients exposed to 5FU or Capecitabine (right). While in the average colorectal metastatic sample the 5FU-related signature contributes close to 2000 mutations, in an average synthetic sample, fewer than 200 5FU-related mutations were injected.

e) Average mutational profile of the 5FU-related signature (mean cosine similarity 0.95 with SBS17b) extracted from each of the 35 groups of 3 synthetic 5FU-exposed samples and 64 AML samples in the WGS AML cohort.

The box in each boxplot delimits the first and third quartiles of the distribution (with a line representing the median); the whiskers delimit the lowest data point above the first quartile minus 1.5 times the interquartile distance and the highest data point below the third quartile plus 1.5 times the interquartile distance.

t-AML, treatment-related AML; 5-FU, 5-fluorouracil; SBS31, platinum-related single base substitution signature; SBS17b, 5-FU-related single base substitution signature.

Supplementary Figure 3

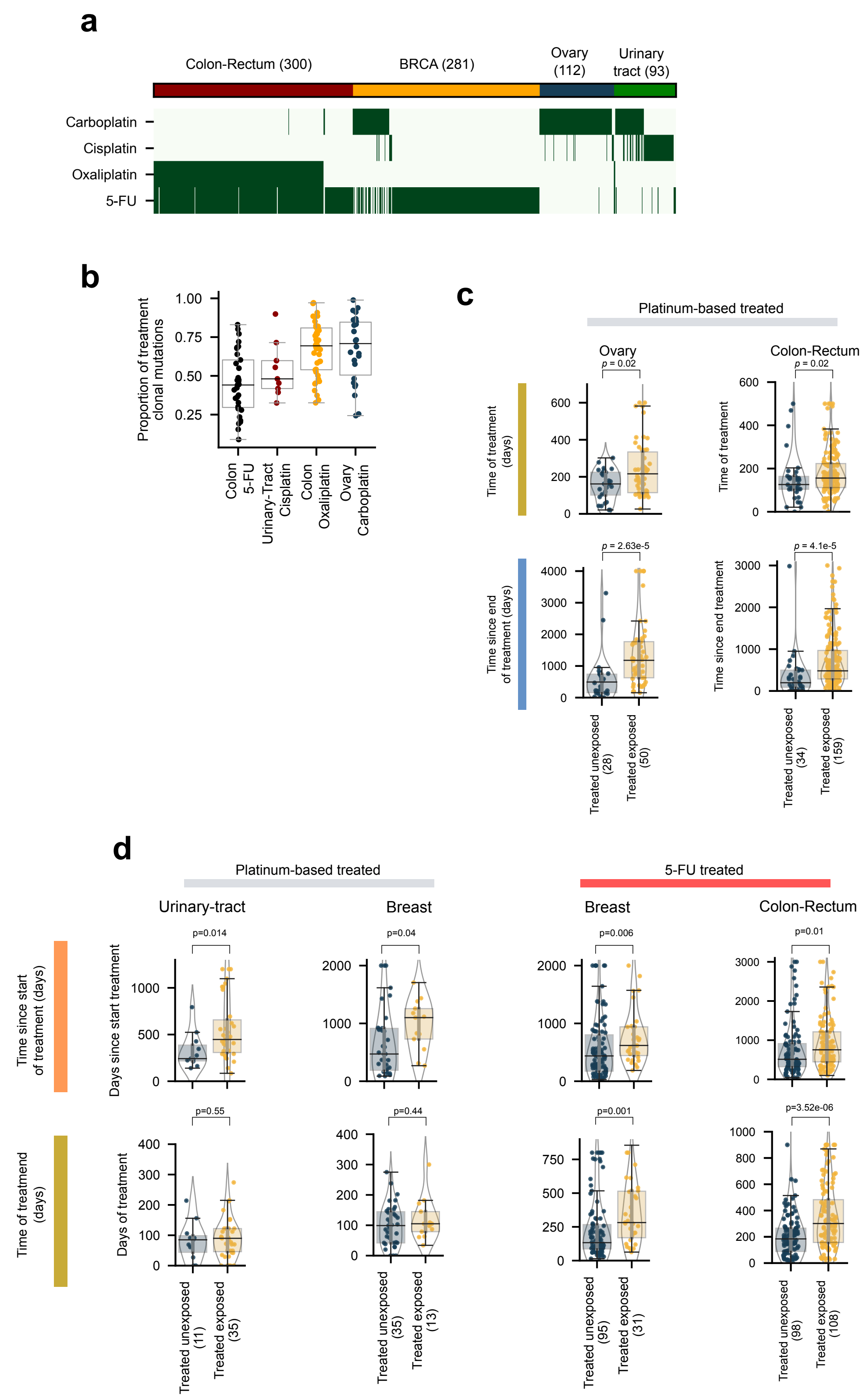

Figure S3. Different mutagenic mechanisms of platinum-based drugs and 5-FU

a) Exposure of metastatic tumors from different organs of origin to platinum-based drugs and 5-FU across the metastasis cohort.

b) Distribution of the proportion of clonal treatment related mutations identified in metastatic tumors from different organs of origin across the metastasis cohort.

c) Distribution of time of treatment (days), time between start of treatment and the biopsy of the metastasis (days) and time between the end of treatment (days) of samples of metastatic tumors of different organs of origin with detectable or undetectable treatment mutations taken from patients exposed to platinum-based drugs or 5-FU. All p-values shown correspond to two-tailed Mann-Whitney tests.

The box in each boxplot delimits the first and third quartiles of the distribution (with a line representing the median); the whiskers delimit the lowest data point above the first quartile minus 1.5 times the interquartile distance and the highest data point below the third quartile plus 1.5 times the interquartile distance.

BRCA, Breast Adenocarcinoma; 5-FU, 5-fluorouracil.

Supplementary Figure 4

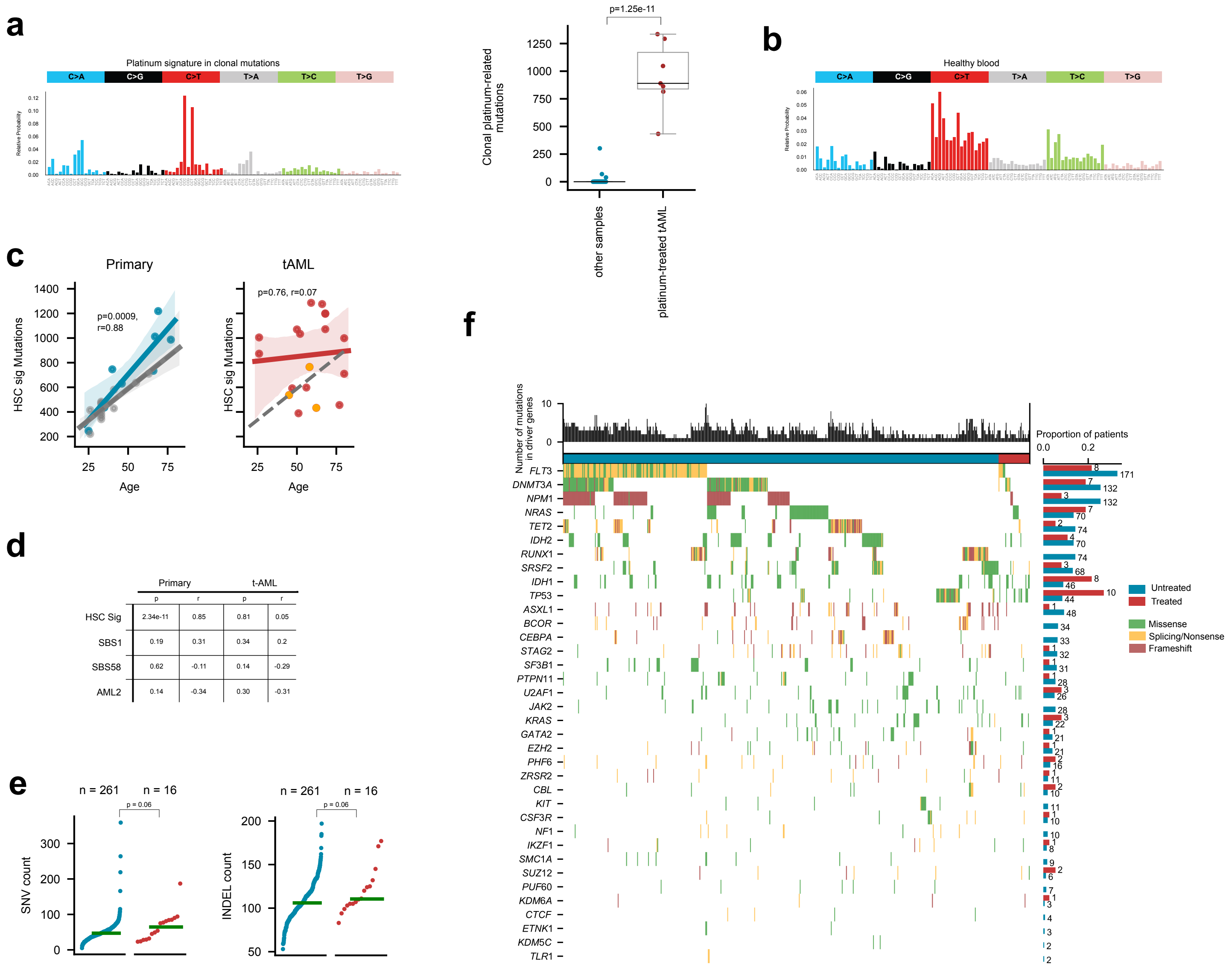

Figure S4. The development of treatment-related AMLs

a) Profile and activity of the platinum-related signature among clonal mutations of tAMLs in the WGS AML cohort. Clonal mutations were identified (Methods) and a de novo extraction of mutational signatures was carried out using only clonal mutations. The significantly higher activity of the platinum-based signature across clonal mutations in platinum-exposed tAMLs (n=7; two-tailed Mann-Whitney test) than across other tAMLs (n=23) confirms that the last clonal expansion that founded the leukemia took place after the beginning of the treatment. The box in the boxplot delimits the first and third quartiles of the distribution (with a line representing the median); the whiskers delimit the lowest data point above the first quartile minus 1.5 times the interquartile distance and the highest data point below the third quartile plus 1.5 times the interquartile distance.

b) Mutational signature of variants accumulated as a result of hematopoiesis cell divisions, across healthy blood samples.

c) Linear relationship between the age of patients and the number of HSC mutations of primary AMLs and tAMLs (as in Fig. 3c of the main paper). In this case, the HSC signature was extracted de novo after removal from the WGS AML cohort of samples with more than 300 mutations contributed by signatures SBS43, SBS58 (potential sequencing artifacts) or AML1 SBS (which may interfere with the HSC signature due to similarity of their most active peaks). The correlation between the number of mutations contributed by the HSC signature resulting from this de novo extraction and the age of patients is still significant (Pearson's correlation coefficient; p=0.0009) as in the case of the HSC extracted from the entire WGS AML cohort (Fig. 3c of the main paper). The shaded areas cover the 95% confidence intervals of the corresponding regression lines.

d) Correlation (Pearson's correlation coefficient and p-value) of the number of mutations contributed by all mutational signatures active in primary and tAML cases in the WGS AML cohort and the age of donors.

e) Comparison (two-tailed Mann-Whitney test) of the number of single nucleotide variants and indels identified in primary AML and tAML cases of the WES AML cohort.

f) Mutations in AML driver genes in primary AML and tAML cases of the WES AML cohort. The total number of mutations in AML driver genes in each AML patient appears as a bar at the top of the heatmap. The total number of mutations in each gene among primary and tAML cases appears represented as two bars at the right side of the heatmap. Primary and tAML cases are separated in the heatmap.

t-AML, treatment-related AML; HSC, Hematopoietic Stem Cell; sig, signature; SNV, Single Nucleotide Variant; INDEL, insertion or deletion.

Supplementary Figure 5

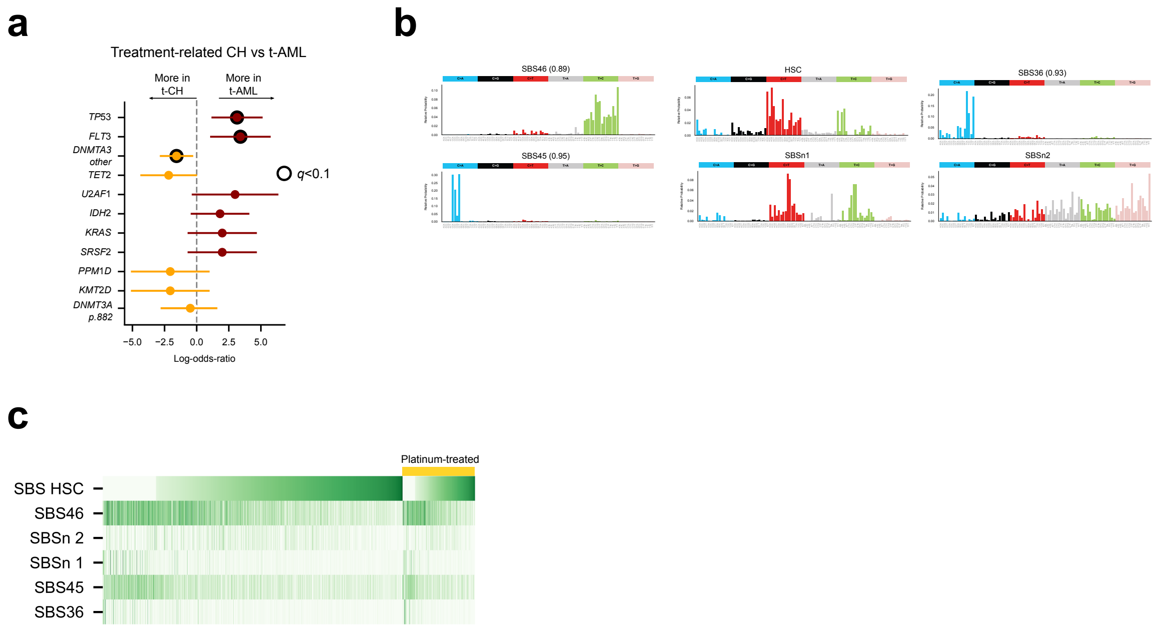

Figure S5. Non-malignant blood samples mutations

a) Overrepresentation of mutations in different genes across CH or tAML cases. Similar to Figure 4b, but separating DNMT3A mutations into two groups depending on whether or not they affect the R882 residue. The bars represent the 95% confidence intervals of the log-odds ratio.

b) Mutational signatures active in the blood samples of the donors of the metastasis cohort. Their names or numbers correspond to the etiology of signatures extracted previously from other cohorts. The numbers coincide with the repertoire of signatures in COSMIC v3.

c) Activity of mutational signatures across blood samples from donors of the metastasis cohort.

CH, Clonal Hemtopoiesis; t-AML, treatment-related AML; HSC, Hematopoietic Stem Cell.

Table S1. The WGS AML cohort

SRA	Patient ID	AML sample ID	Germline sample ID	Treatment	Primary Location	Project	Age	DBS	INDELS	SNVs
SRR1802602_SRR1802367	180866	SRR1802602	SRR1802367	Alkylating Drug,Proteasome Inhibitor,Radiation	Multiple Myeloma	phs000159	66	35	533	2177
SRR388810_SRR388850	189941	SRR388810	SRR388850	Platinum-based Drug,Microtubule Inhibitor,Radiation	Breast/Ovarian	phs000159	42	55	264	3755
SRR1801385_SRR1800351	266608	SRR1801385	SRR1800351	Nucleoside Metabolic Inhibitor	Renal	phs000159	80	43	712	1599
SRR1801780_SRR1800379	400992	SRR1801780	SRR1800379	Anthracycline Topoisomerase Inhibitor,Alkylating Drug,Radiation	Breast	phs000159	52	21	531	2256
SRR1801924_SRR1802263	476081	SRR1801924	SRR1802263	Anthracycline Topoisomerase Inhibitor,Alkylating Drug,Radiation	Breast	phs000159	68	29	636	2087
SRR1801916_SRR1800363	476204	SRR1801916	SRR1800363	Unknown,Radiation	Breast	phs000159	51	23	490	1142
SRR1802114_SRR1800784	514901	SRR1802114	SRR1800784	Radiation	Breast	phs000159	63	29	627	875
SRR1802086_SRR1800793	548417	SRR1802086	SRR1800793	Unknown	Breast	phs000159	77	21	571	1258
SRR1801954_SRR1800335	572162	SRR1801954	SRR1800335	Anthracycline Topoisomerase Inhibitor,Alkylating Drug,Radiation	Breast	phs000159	59	24	494	2166
SRR1803002_SRR1802357	596616	SRR1803002	SRR1802357	Topoisomerase Inhibitor	Multiple Sclerosis	phs000159	43	35	2053	2073
SRR1801946_SRR1801393	597808	SRR1801946	SRR1801393	Anthracycline Topoisomerase Inhibitor,Alkylating Drug,Radiation	Breast	phs000159	59	21	403	2239
SRR1802102_SRR1800371	644242	SRR1802102	SRR1800371	Anthracycline Topoisomerase Inhibitor,Alkylating Drug,Microtubule Inhibitor,Radiation	Breast	phs000159	56	33	484	1504
SRR1802629_SRR1802255	658208	SRR1802629	SRR1802255	Topoisomerase Inhibitor	Multiple Sclerosis	phs000159	50	39	692	2090
SRR1802637_SRR1802586	698494	SRR1802637	SRR1802586	Anthracycline Topoisomerase Inhibitor,Microtubule Inhibitor	Non-Hodgkin's Lymphoma	phs000159	57	21	723	2093
SRR1802228_SRR1800776	706395	SRR1802228	SRR1800776	Platinum-based Drug,Anthracycline Topoisomerase Inhibitor,Topoisomerase Inhibitor,Radiation	Lung	phs000159	45	41	566	2193
SRR1802338_SRR1801402	717044	SRR1802338	SRR1801402	Unknown,Radiation	Uterine	phs000159	80	19	532	2083
SRR1802695_SRR1802564	779828	SRR1802695	SRR1802564	Radiation	Prostate	phs000159	68	24	489	2272
SRR1802703_SRR1802556	811184	SRR1802703	SRR1802556	Anthracycline Topoisomerase Inhibitor,Microtubule Inhibitor	Non-Hodgkin's Lymphoma	phs000159	26	33	465	1953
SRR1802798_SRR1802439	856024	SRR1802798	SRR1802439	Anthracycline Topoisomerase Inhibitor,Microtubule Inhibitor,Radiation	Non-Hodgkin's Lymphoma	phs000159	26	33	464	1946
SRR1802711_SRR1802494	864484	SRR1802711	SRR1802494	Topoisomerase Inhibitor,Radiation,Unknown	Testicular	phs000159	39	93	1615	5388
SRR1802754_SRR1802621	925964	SRR1802754	SRR1802621	Platinum-based Drug,Microtubule Inhibitor,Topoisomerase Inhibitor,Nucleoside Metabolic Inhibitor	Ovarian	phs000159	58	33	758	2160
SRR1802790_SRR1802664	982895	SRR1802790	SRR1802664	Radiation	Breast	phs000159	47	20	405	1394
SRR390572_SRR390546	461282	SRR390572	SRR390546	Unknown	Prostate/Pancreatic cancer	phs000159	70	14	928	3022
SRR523202_SRR523754	400220	SRR523202	SRR523754	Unknown	nan	phs000159	34	20	495	1614
SRR767415_SRR767463	838538	SRR767415	SRR767463	Unknown	Kidney	phs000159	67	22	387	2799
AP0388_AP0389	INHOUSE_1	AP0388	AP0389	Platinum-based Drug,Radiation	Head and Neck	In-house	62.3	44	517	2081
AP0394_AP0391	INHOUSE_2	AP0394	AP0391	Nucleoside Metabolic Inhibitor,Radiation	Colon-Rectum/Endometrium	In-house	68	25	846	2356
AP0390_AP0395	INHOUSE_3	AP0390	AP0395	Nucleoside Metabolic Inhibitor,Platinum-based Drug,Anthracycline Topoisomerase Inhibitor,Microtubule Inhibitor,Radiation	Breast/Ovarian	In-house	49.8	72	815	2834
18b07e20-f8f3-11e6-942e-3c4a9275d6c8_81ab3998-c917-11e7-bf6c-3c4a9275d6c8	WTS1	18b07e20-f8f3-11e6-942e-3c4a9275d6c8	81ab3998-c917-11e7-bf6c-3c4a9275d6c8	Platinum-based Drug	Breast/Ovarian	EGAD00001005028	NA	36	1662	3073
4bfc776e-99ea-11e7-b9f0-3c4a9275d6c8_4bf5aef2-99ea-11e7-b9f0-3c4a9275d6c8	WTS2	4bfc776e-99ea-11e7-b9f0-3c4a9275d6c8	4bf5aef2-99ea-11e7-b9f0-3c4a9275d6c8	Platinum-based Drug	Breast/Ovarian	EGAD00001005028	NA	66	3295	3011

Table S2. Logistic regression model for metastatic tumors exposed to platinum-based drugs (top) and 5-FU (bottom)

Formula	BiC	AUC-ROC
exp ~ 1	347.87	0.50
exp ~ 1 + days_treat	348.21	0.61
exp ~ 1 + days_since_end	332.36	0.71
exp ~ 1 + prox_v	342.76	0.60
exp ~ 1 + days_treat + days_since_end + prox_v	329.96	0.71
exp ~ 1 + days_treat + days_since_end + prox_v + tumor_type	329.18	0.73
exp ~ 1 + days_treat:tumor_type + days_since_end + prox_v	336.63	0.72
exp ~ 1 + days_treat + days_since_end:tumor_type + prox_v	330.61	0.73
exp ~ 1 + days_treat:tumor_type + days_since_end:tumor_type + prox_v	341.89	0.72
exp ~ 1 + days_treat*tumor_type + days_since_end:tumor_type + prox_v	348.85	0.72
exp ~ 1 + days_treat:tumor_type + days_since_end*tumor_type + prox_v	348.85	0.72
exp ~ 1 + days_treat*tumor_type + days_since_end*tumor_type + prox_v	348.85	0.72
exp ~ 1 + days_treat*tumor_type + days_since_end*tumor_type + prox_v*tumor_type	360.06	nan
exp ~ 1 + days_treat*days_since_end*tumor_type + prox_v	353.00	nan

Formula	BiC	AUC-ROC
exp ~ 1	457.23	0.50
exp ~ 1 + days_treat	447.49	0.69
exp ~ 1 + days_since_end	459.46	0.59
exp ~ 1 + prox_v	451.35	0.58
exp ~ 1 + days_treat + days_since_end + prox_v	447.48	0.69
exp ~ 1 + days_treat + days_since_end + prox_v + tumor_type	433.63	0.71
exp ~ 1 + days_treat:tumor_type + days_since_end + prox_v	434.12	0.71
exp ~ 1 + days_treat + days_since_end:tumor_type + prox_v	440.82	0.70
exp ~ 1 + days_treat:tumor_type + days_since_end:tumor_type + prox_v	439.09	0.70
exp ~ 1 + days_treat*tumor_type + days_since_end:tumor_type + prox_v	442.14	0.71
exp ~ 1 + days_treat:tumor_type + days_since_end*tumor_type + prox_v	442.14	0.71
exp ~ 1 + days_treat*tumor_type + days_since_end*tumor_type + prox_v	442.14	0.71
exp ~ 1 + days_treat*tumor_type + days_since_end*tumor_type + prox_v*tumor_type	446.08	0.70

Table S3. AML driver genes and their mutations across cases in the WXS cohort.

SYMBOL	METHODS	QVALUE INTOGEN	MUTATED SAMPLES	TRANSCRIPT	SAMPLES	RATE MUTATED SAMPLES	EXCESS MISSENSE MUTATIONS	EXCESS NONSENSE MUTATIONS	EXCESS SPLICING MUTATIONS	IN CGC	DOMAIN ENRICHED FOR MUTATIONS	LINEAR CLUSTERS COORDINATES	POSITION 3D CLUSTERS MUTATIONS
ASXL1	cbase	9.63E-05	14	ENST00000375687	277	0.051	0.00	0.99	0.00	True			
BCOR	oncodrivefml,dndscv,cbase	9.31E-19	11	ENST00000378444	277	0.040	0.51	0.99	0.99	True			
CBL	oncodrivefml,smregions,cbase	2.17E-08	5	ENST00000264033	277	0.018	0.93	0.99	0.99	True	PF14447:381:429		
CEBPA	oncodrivefml,dndscv,cbase	2.96E-18	14	ENST00000498907	277	0.051	0.00	1.00	0.00	True			
CSF3R	oncodrivefml,dndscv,cbase	2.43E-17	6	ENST00000373103	277	0.022	0.96	0.99	0.00	True		36467833:36467833	
CTCF	combination	2.19E-03	2	ENST00000646076	277	0.007	0.90	0.00	0.99	True			
DNMT3A	oncodrivefml,oncodriveclustl,dndscv,cbase,mutpanning,hotmaps	1.11E-41	34	ENST00000264709	277	0.123	0.99	0.99	0.99	True		25234373:25234383	882
ETNK1	oncodriveclustl	3.92E-02	2	ENST00000266517	277	0.007	0.94	0.00	0.00	True		22659061:22659061	
EZH2	oncodrivefml,dndscv,cbase	2.47E-08	11	ENST00000320356	277	0.040	0.98	0.98	0.00	True			
FLT3	v,smregions,cbase,mutpanning,hotmaps	1.49E-34	24	ENST00000241453	277	0.087	0.99	0.00	0.00	True	PF07714:610:943	28018485:28018505	839835
GATA2	oncodrivefml,dndscv,smregions,cbase,mutpanning	1.68E-24	10	ENST00000341105	277	0.036	0.99	0.99	0.00	True	PF00320:295:328		
IDH1	oncodriveclustl,dndscv,cbase,mutpanning,hotmaps	1.18E-31	21	ENST00000415913	277	0.076	1.00	0.00	0.00	True		208248388:208248389	132
IDH2	v,smregions,cbase,mutpanning,hotmaps	4.43E-44	31	ENST00000330062	277	0.112	1.00	0.00	0.00	True	PF00180:45:441	90088702:90088702	172140
IKZF1	oncodrivefml,oncodriveclustl,dndscv,smregions,cbase	8.73E-17	6	ENST00000644005	277	0.022	0.96	0.99	0.00	True	PF00096:145:167	50382594:50382594	
JAK2	oncodriveclustl,dndscv,smregions,mutpanning,hotmaps	9.35E-12	6	ENST00000381652	277	0.022	0.97	0.00	0.00	True	PF07714:546:805	5073770:5073770	617
KDM5C	combination	6.50E-02	2	ENST00000375401	277	0.007	0.81	0.00	0.00	True			
KDM6A	combination	5.98E-03	2	ENST00000377967	277	0.007	0.00	0.95	0.00	True			
KIT	cbase	1.38E-03	3	ENST00000288135	277	0.011	0.89	0.98	0.00	True			
KRAS	dndscv,cbase,hotmaps	2.42E-05	8	ENST00000256078	277	0.029	1.00	0.00	0.00	True			61,13
NF1	oncodrivefml,oncodriveclustl,cbase	3.81E-13	7	ENST00000358273	277	0.025	0.86	0.98	0.00	True		31214536:31214536	
NPM1	oncodrivefml,mutpanning	1.51E-17	51	ENST00000296930	277	0.184	0.00	0.00	0.00	True			
NRAS	oncodriveclustl,dndscv,smregions,cbase,mutpanning,hotmaps	1.68E-24	27	ENST00000369535	277	0.097	1.00	0.00	0.00	True	PF00071:5:164	114716123:114716127	13,12,61
PHF6	oncodrivefml,dndscv,smregions,cbase	9.06E-19	10	ENST00000332070	277	0.036	0.98	1.00	0.00	True	PF13771:239:330	134417289:134417289	
PTPN11	v,smregions,cbase,mutpanning,hotmaps	6.32E-19	15	ENST00000635625	277	0.054	0.99	0.00	0.00	True	PF00017:6:81	112450395:112450407,112489081:112489084	61,60,285,76,507,72,506
PUF60	dndscv,cbase	6.63E-05	6	ENST00000526683	277	0.022	0.98	0.00	0.00	False			
RUNX1	oncodrivefml,dndscv,smregions,cbase,mutpanning,hotmaps	4.81E-34	30	ENST00000300305	277	0.108	0.99	1.00	1.00	True	PF00853:79:206		107
SF3B1	oncodriveclustl,dndscv,cbase,mutpanning	5.66E-11	11	ENST00000335508	277	0.040	0.97	0.00	0.00	True		197402635:197402647	
SMC1A	oncodriveclustl,dndscv,cbase,hotmaps	2.64E-06	8	ENST00000322213	277	0.029	0.97	0.00	0.00	True		53414992:53414992,53403617:53403617	
SRSF2	oncodriveclustl,dndscv,cbase,mutpanning,hotmaps	1.08E-34	18	ENST00000392485	277	0.065	0.99	0.00	0.00	True		76736877:76736877	95
STAG2	oncodrivefml,dndscv,cbase	2.17E-08	10	ENST00000218089	277	0.036	0.71	0.99	0.99	True			
SUZ12	oncodrivefml,cbase	2.42E-05	3	ENST00000322652	277	0.011	0.92	0.98	0.00	True			
TET2	oncodrivefml,dndscv,cbase,mutpanning	1.96E-16	22	ENST00000540549	277	0.079	0.97	1.00	1.00	True			
TLR1	oncodrivefml,dndscv,cbase	1.84E-06	2	ENST00000308979	277	0.007	0.00	1.00	0.00	False		38796603:38796603	
TP53	oncodrivefml,dndscv,smregions,cbase,mutpanning,hotmaps	3.16E-28	18	ENST00000269305	277	0.065	0.99	1.00	1.00	True	PF00870:95:288	7674210:7674221	1.7327325E+26
U2AF1	oncodrivefml,oncodriveclustl,dndscv,smregions,cbase,mutpanning	1.50E-28	16	ENST00000291552	277	0.058	0.99	0.00	0.00	True	PF00642:15:38,PF00642:150:174	43094667:43094676,43104346:43104346	
ZRSR2	dndscv	9.97E-03	3	ENST00000307771	277	0.011	0.00	0.99	0.99	True			

The evolution of hematopoietic cells under cancer therapy

Supplementary Note

Contents

Supplementary Note 0: Abstract **2**

Supplementary Note 1: Summary **3**

Supplementary Note 2: Synthetic data **4**

Background-Stochastic Samples 4

Background-Observed Samples 5

Injecting Foreign Mutations 5

Synthetic Catalogues of Mutations 6

Supplementary Note 3: Foreign Signature Analysis **6**

Signature Detection Method 6

Observed Samples by Treatment Regimen 7

Synthetic Samples 8

Supplementary Note 4: Discussion **9**

Supplementary Note 0: Abstract

Assuming that the clonal expansion giving rise to clonal hematopoiesis (CH) starts after the beginning of the exposure to a treatment (e.g., platinum-based drugs), treatment-related mutations and hematopoiesis mutations in the cell founding the CH would appear at the same level of clonality in a blood sample. Therefore, if hematopoiesis mutations are detected in this sample, we reasoned that we expect to also detect treatment-related mutations if these appear in numbers that rise above the limit of detection of mutational profile reconstruction methods. This may be divided into two questions:

i) What is the limit of detection (in number of mutations contributed by a given signature) of mutational profile reconstruction methods?

ii) Do we reasonably expect a number of treatment-related mutations in CH cases that is above this limit?

To answer the first question, we set up a simulation experiment described in this Supplementary Note. First, we generated synthetic blood samples with the same mutational burden and mutational profile as those observed across blood samples (accounting for hematopoiesis and other mutational signatures identified in the cohort). We call this the *mutational background* of blood samples. Then, we injected in this background across samples an increasing number of mutations following sequence preferences derived from the tri-nucleotide frequencies of platinum-related signatures. In each of these experiments we tested whether the reconstructed mutational profile of the background cohort was significantly different from that of the cohort with treatment mutations (comprising increasing numbers of injected mutations), as measured by a state-of-the-art method (mSigAct).

The experiments demonstrate that for an average number of treatment-related mutations higher than or equal to 10, the mSigAct can effectively separate cohort-wise platinum-related mutational activity from the mutational background. We therefore concluded that the limit of detection of the method is 10 for platinum-related mutations.

Supplementary Note 1: Summary

When detecting the activity of a mutational process in the catalogue of mutations of a cohort (e.g., signature induced by platinum-based chemotherapy) we can in general resort to signature deconstruction or fitting and statistical testing thereupon, from which we can also infer the activity sample-wise. However, these methods generally require a sufficiently high mutation count and cohort size to render a clear signal-to-noise.

In our study we were interested in conducting signature detection with the requirement to handle low mutation counts: low counts preclude a straightforward interpretation sample by sample because of the high false discovery rate and reduced statistical power.

To circumvent this limitation, we laid out a computational analysis to detect the activity of a foreign signature at the cohort level by applying a signature detection method sample by sample and then pooling the results. The rationale is to compare distributions of reconstructed exposures to reveal latent signature activities in the cohort, even if these activities cannot be confidently distributed across samples.

We generated synthetic catalogues of mutations with/without activity of a specific foreign signature of interest. The approach resorted to an abstraction of the common mutational patterns in our cohort of study.

Next we interrogated the observed and simulated catalogues with a state-of-the-art signature detection method that yields as a result a reconstructed exposure to the foreign signature of interest and a significance level accounting for the reconstruction improvement if the foreign signature is included [1].

With these outputs we compared the observed and simulated catalogues, thereby assessing the activity of specific mutational processes in a cohort of samples and empirically validating the sensitivity and specificity of the signature detection method in our setting. We hereto describe the methodological steps in detail and report our findings.

Supplementary Note 2: Synthetic data

We followed closely the simulation approach laid out in [1]. We generated synthetic mutation data (single base substitution) by the addition of two catalogues of mutations: i) background mutations representative of the mutational profile of samples from untreated donors; ii) foreign (chemotherapy-associated) mutations. Here the term *catalogue of mutations* stands for a tabular data set of mutation counts across the 96 possible trinucleotide contexts comprising all possible single base substitutions with a pyrimidine reference allele.

Background-Stochastic Samples

The catalogue of mutations obtained from blood samples of untreated donors of the metastasis cohort (i.e. not subject to any treatment regimen by the time of collection) was taken as the reference for the background mutational signal. The starting point is to provide a (96 channel) signature deconstruction of this catalogue. Given the signature deconstruction of the reference catalogue of mutations, let π_S be the proportion of samples with non-zero exposure for each active signature S in the deconstruction. Also, for each signature we can compute the best gamma distribution fitting of non-zero exposures reported for S. We denote the resulting gamma distribution $\mathbf{Gamma}(\alpha_S, \beta_S)$. The choice of the gamma distribution is justified in order to capture the skewness of the non-zero exposures.

We then use π_S and $\mathbf{Gamma}(\alpha_S, \beta_S)$ computed for each signature S to draw synthetic samples. First, we randomly decide which signatures will remain active, independently from each $\mathbf{Bernoulli}(\pi_S)$. Then for each active signature S we draw its exposure e_S from $\mathbf{Gamma}(\alpha_S, \beta_S)$. The non-active signatures do not contribute any mutations to the catalogue.

The mutation count attributed to S in context c is drawn independently from a negative binomial distribution $\mathbf{NegBinom}(\mu_{S,c}, \sigma)$, with mean $\mu_{S,c}$ and overdispersion parameter $\sigma = 0.1$. The parameter $\mu_{S,c} = e_S \cdot f_{S,c}$ is the mean mutation count generated by signature S in channel c, obtained as the product of the exposure of S and the frequency $f_{S,c}$ of context c in the profile of signature S. Adding the catalogues generated for all active signatures at each of the 96 contexts, we obtain the catalogue of a synthetic sample.

This procedure to model a typical blood sample from an untreated donor in the metastatic cohort, from which we can randomly draw synthetic catalogues of mutations, has also the practical advantage that it effectively filters spurious signals that may be active in a subset of samples, but are not accounted for by any of the signatures employed in the deconstruction.

Background-Observed Samples

We were also interested in generating synthetic using as a background the observed catalogue of mutations of blood samples from untreated donors of the metastasis cohort.

In sum, we used two sets of mutational catalogues for subsequent simulation: so-called background-stochastic (4,000 randomly generated catalogues, 1,000 samples independently drawn for each specific foreign signature analysis) and background-observed (1,038 observed catalogues from blood samples of untreated donors).

Injecting Foreign Mutations

For the rest of our analysis, we probed 4 previously reported single base substitution signatures associated with platinum-based and 5-FU-based chemotherapy regimens: i) signature E-SBS17b [2] (cosine similarity 0.97 to COSMIC SBS17b), associated to 5-fluorouracil; ii) a signature associated with oxaliplatin; COSMIC (v3.0), iii and iv) signatures SBS31 and SBS35, associated with platinum-based chemotherapy regimens [2, 3]. For this supplementary note, we will label these signatures as: "SBS17b", "oxaliplatin", "SBS31" and "SBS35", respectively.

Synthetic data sets are the result of adding a background catalogue and a foreign catalogue generated from the foreign signature of interest at a specified level of exposure per sample: foreign signatures are thus "injected" in the background catalogue. In a similar fashion as described above, if we denote the foreign signature as S, injecting S at a level of exposure e_S implies to add as many counts in context c as drawn from $\mathbf{NegBinom}(\mu_{S,c}, \sigma)$, the negative binomial distribution with mean $\mu_{S,c} = e \cdot f_{S,c}$ and overdispersion $\sigma = 0.1$, where $f_{S,c}$ denotes the frequency of context c in the profile of signature S.

Synthetic Catalogues of Mutations

For each signature S out of the 4 possible chemotherapy-associated signatures (3 platinum-related and 1 5-FU-related) and for each level of exposure in a probing grid

$$\mathcal{G} = \{2, 5, 7, 10, 15, 20, 25, 30, 35, 40, 45, 50, 75, 100\}$$

a synthetic catalogue was generated by taking a background catalogue, then injecting the foreign signature at that level of exposure value. This procedure was carried out for all the background-stochastic and the background-observed catalogues.

Supplementary Note 3: Foreign Signature Analysis

Signature Detection Method

To test for the presence of a mutational signature in a specific sample we used the method so-called `mSigAct` [1]. Given a set of signatures deemed to explain the mutations of the sample (background), the method tests whether the inclusion of an additionally provided signature (foreign) improves the reconstruction significantly.

Specifically, the method models the mutation count data as being negative binomial distributed and carries out a likelihood-ratio test comparing the likelihood of the observed catalogue under two models: with/without the foreign signature. Assuming one of the models hold and we are able to estimate the exposures to each signature that best explain a sample, for each trinucleotide context c we have an actual mutation count m_c and a reconstructed mutation count r_c. The log-likelihood of the actual catalogue is then computed as

$$\mathcal{L} = \sum_c \log P(m_c \mid r_c, \sigma),$$

where P is the negative binomial probability of m_c, governed by the mean r_c, with σ being an appropriate overdispersion estimate.

The reconstructed mutation count parameters r_c depend on the estimation of the exposures for the signatures considered in the model (with/without foreign signature). These exposures are obtained upon non-linear optimization, with the likelihood $\mathcal{L}$ acting as objective function. For a more detailed exposition, the reader is encouraged to go through the Supplementary Materials and Methods of [1].

Given the background and foreign signatures, the output of `mSigAct` provides a reconstructed exposure for the foreign signature alongside the significance witnessed by the likelihood-ratio test. Both the synthetic samples derived from the background-stochastic and from the background-observed, including the background catalogues themselves (with zero mutations injected) were subject to `mSigAct` analysis.

Observed Samples by Treatment Regimen

We segregated the samples from the metastasis cohort into groups by treatment regimen. For each foreign signature considered we compared the distributions of the most likely reconstructed exposures of the foreign signature upon `mSigAct` analysis and compared the distributions between treatment groups and the background group of untreated samples.

We focused on five treatment regimens: carboplatin, cisplatin, oxaliplatin (platin-based); fluorouracil and capecitabine (nucleoside metabolic inhibitors or NMI). For each treatment we conducted treated/untreated comparisons for each foreign signature, thereby interrogating whether the treatment has any discernible effect at the cohort level in terms of the foreign signature activity. For each comparison we carried out a Kolmogorov-Smirnov (KS) two-sample test to render a significance value for the discrepancy between distributions (see Supp. Fig. 6 of which the main Figure 6e is the SBS31-cisplatin case). None of the comparisons provided any evidence of significant differences between treated/untreated groups for any of the treatment-associated signatures considered.

We then carried out the same exercise with the distributions of p-values upon `mSigAct` analysis. When taking all samples into account, the analysis produced no differences between treated and untreated samples. Then we reasoned that we might still be able to identify a subgroup of samples with a

signature-treatment effect if we compared only samples with non-zero reconstructed exposure. We carried out this analysis with different thresholds. In Supp. Fig. 7 we show the result for samples with exposure ≥ 5. None of the comparisons yielded any significant differences between treated/untreated groups for any of the treatment-associated signatures considered. Remark that in one case (signature SBS31 and cisplatin) the comparison showed a weak although non-significant trend favouring the hypothesis that the treated samples bore a footprint of SBS31.

Synthetic Samples

We also analyzed the synthetic samples with known levels of exposure to injected signatures, including the background (zero-injected samples). This analysis allowed us to have a more concrete understanding of what signal-to-noise ratios are expected when considering different levels of true exposure to the signature. In other words, considering the previous comparisons, is it possible that at the expected levels of exposure, the treatment-signature association signal was too weak for our statistical procedure to detect it?

For each signature and each level of injection a KS two-sample test was carried out comparing either the exposure or p-value distribution of the injected synthetic samples injected to the background. We did it in the two settings derived from background-observed (Supp. Fig. 8) and from background-stochastic samples (Supp. Fig. 9), respectively.

As expected, in both synthetic data sets the signal-to-noise was dependent on the signature of interest. For example, SBS17b showed the strongest effects, both in terms of reconstructed exposures and p-values, even with few injected mutations. On the opposite side, the signal-to-noise induced by SBS31 injection did not reach KS significance ($p < 0.01$) below the level of 7 injected mutations. That said, for all signatures and all levels of exposures, the same trend is apparent.

Interestingly, when comparing the results between both synthetic settings, we see that the false-positive rate (i.e., proportion of non-zero exposure reconstructed from zero-injected samples) is lower and more stable in the background-stochastic catalogue (~ 0.025) than in the background-observed catalogue, where this rate fluctuates between ~ 0.125 (SBS35) and ~ 0.025 (SBS17b).

Supplementary Note 4: Discussion

In our analysis we compared treated/untreated samples, then conducted the same signature detection with synthetic samples in two different settings. None of the treated/untreated comparisons rendered significant differences nor trends suggestive of an effect. However with the same tools we were able to identify a clear signal-to-noise in the synthetic data sets, demonstrating that the lack of signal in the first analysis is not due to a technical limitation, but rather to the absence of activity of the foreign signatures.

Only in one case (signature SBS31 and cisplatin) we found a non-significant trend consistent with a small subset of samples having this treatment-signature association.

The analyses and results presented in this supplementary note are highly consistent with the conclusion that there are no homogeneous treatment-signature effects for the signatures and treatments considered across healthy blood samples from patients of the metastasis cohort.

References

[1] A. W. T. Ng, S. L. Poon, M. N. Huang, J. Q. Lim, A. Boot, W. Yu, Y. Suzuki, S. Thangaraju, C. C. Y. Ng, P. Tan, S. T. Pang, H. Y. Huang, M. C. Yu, P. H. Lee, S. Y. Hsieh, A. Y. Chang, B. T. Teh, and S. G. Rozen. Aristolochic acids and their derivatives are widely implicated in liver cancers in Taiwan and throughout Asia. *Sci Transl Med*, 9(412), Oct 2017.

[2] O. Pich, F. Muiños, M. P. Lolkema, N. Steeghs, A. Gonzalez-Perez, and N. Lopez-Bigas. The mutational footprints of cancer therapies. *Nat. Genet.*, 51(12):1732–1740, 12 2019.

[3] L. B. Alexandrov, J. Kim, N. J. Haradhvala, M. N. Huang, A. W. Tian Ng, Y. Wu, A. Boot, K. R. Covington, D. A. Gordenin, E. N. Bergstrom, S. M. A. Islam, N. Lopez-Bigas, L. J. Klimczak, J. R. McPherson, S. Morganella, R. Sabarinathan, D. A. Wheeler, V. Mustonen, G. Getz, S. G. Rozen, M. R. Stratton, L. B. Alexandrov, E. N. Bergstrom, A. Boot, P. Boutros, K. Chan, K. R. Covington, A. Fujimoto, G. Getz, D. A. Gordenin, N. J. Haradhvala, M. N. Huang, S. M. A. Islam, M. Kazanov, J. Kim, L. J. Klimczak, N. Lopez-Bigas, M. Lawrence, I. Martincorena, J. R. McPherson, S. Morganella, V. Mustonen, H. Nakagawa, A. W. Tian Ng, P. Polak, S. Prokopec, S. A. Roberts, S. G. Rozen, R. Sabarinathan, N. Saini, T. Shibata, Y. Shiraishi, M. R. Stratton, B. T. Teh, I. Vazquez-Garcia, D. A. Wheeler, Y. Wu, F. Yousif, and W. Yu. The repertoire of mutational signatures in human cancer. *Nature*, 578(7793):94–101, 02 2020.

Supplementary Figure 6: Cumulative densities of reconstructed exposures obtained with `mSigAct` from samples of the metastasis cohort. Plots show the cumulative density of the reconstructed exposure distribution of treated versus untreated samples for each treatment (row) and candidate signature (column).

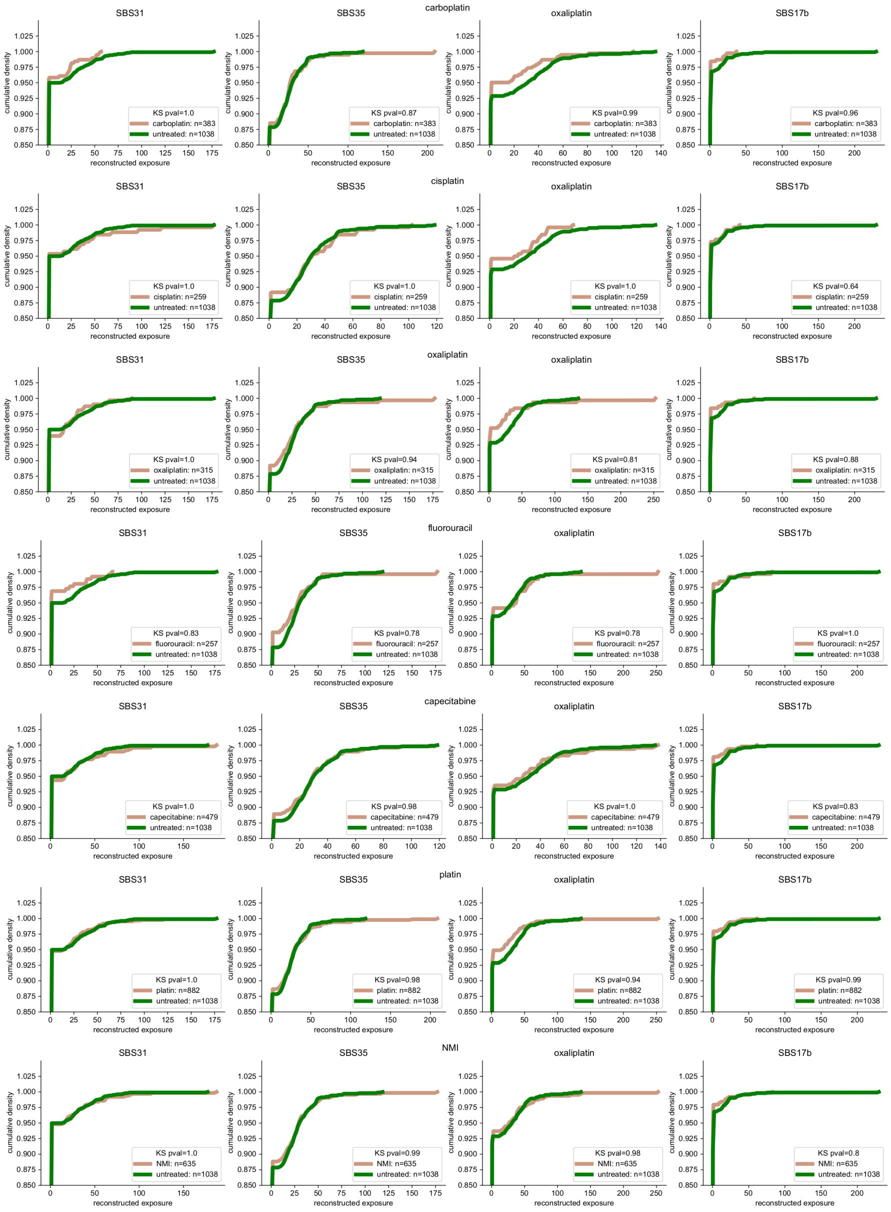

Supplementary Figure 7: Cumulative densities of reconstructed p-values obtained with `mSigAct` from samples with reconstructed exposure ≥ 5. Plots show the cumulative density of the reconstructed exposure distribution of treated versus untreated samples for each treatment (row) and candidate signature (column).

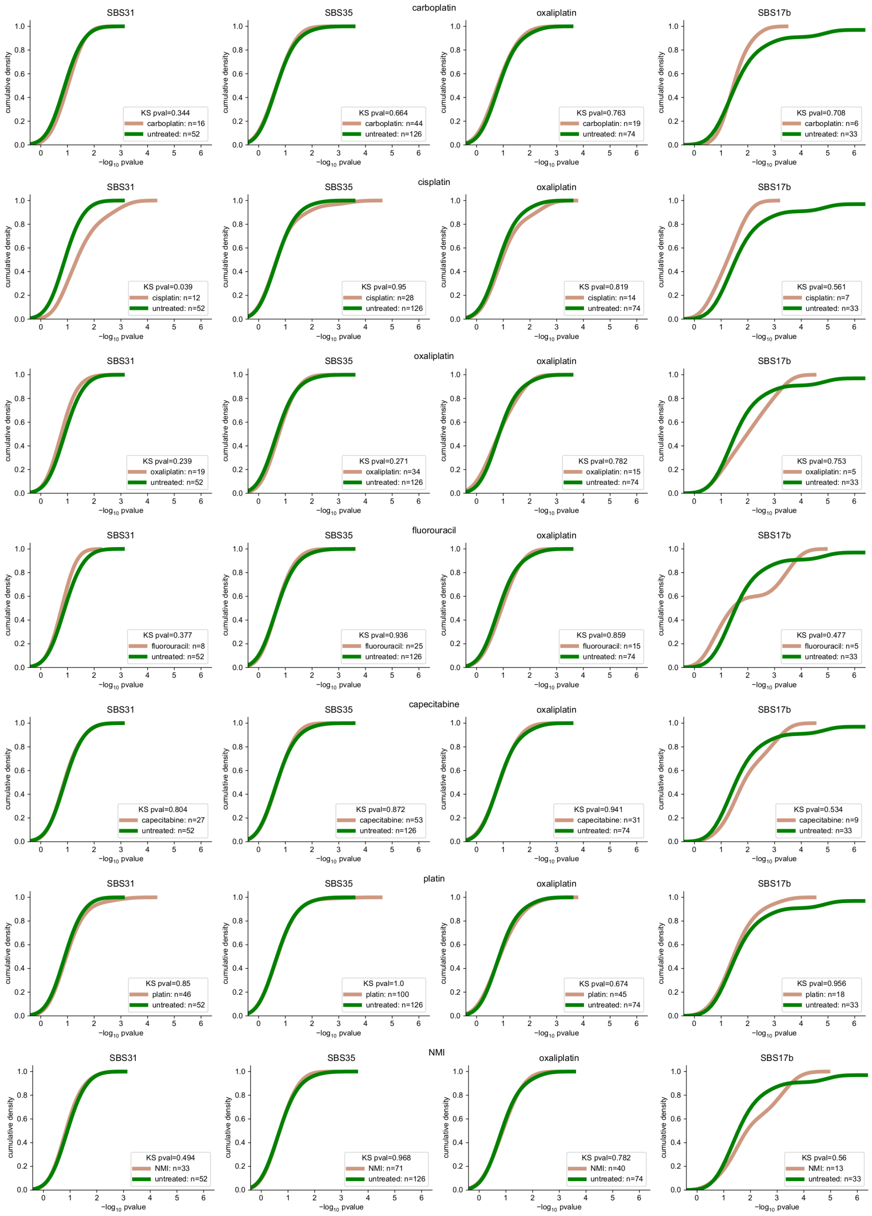

Supplementary Figure 8: Cumulative density functions of the reconstructed exposures (Panel a) and p-values (Panel b) after running `mSigAct` on the synthetic data set derived from the background-observed samples.

a

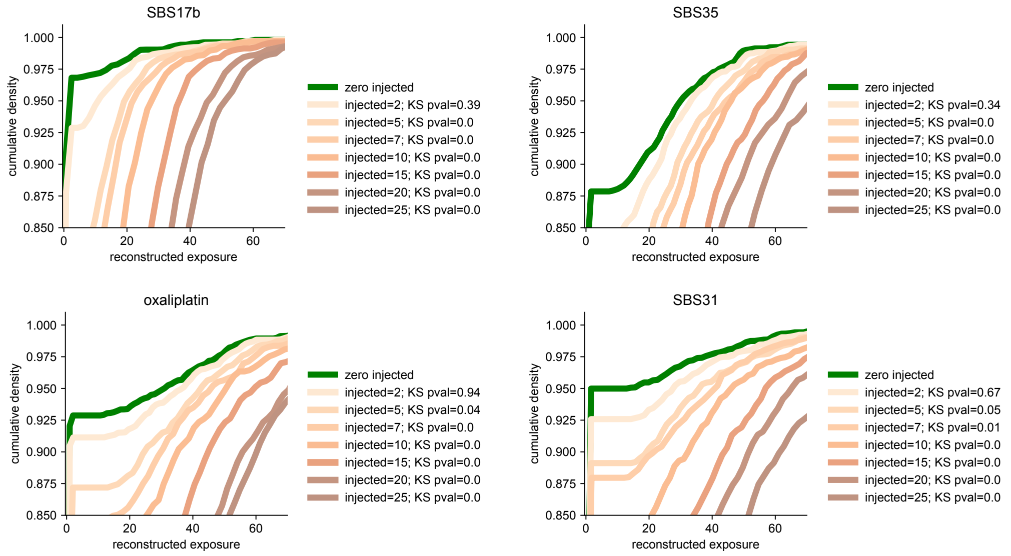

b

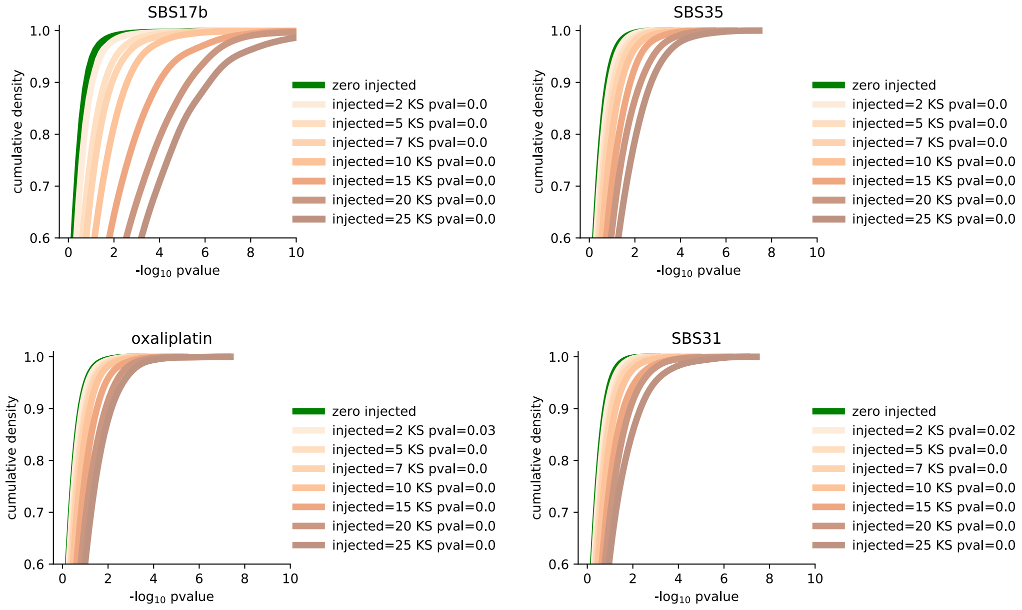

Supplementary Figure 9: Cumulative density functions of the reconstructed exposures (Panel a) and p-values (Panel b) after running `mSigAct` on the synthetic data set derived from the background-stochastic samples.

a

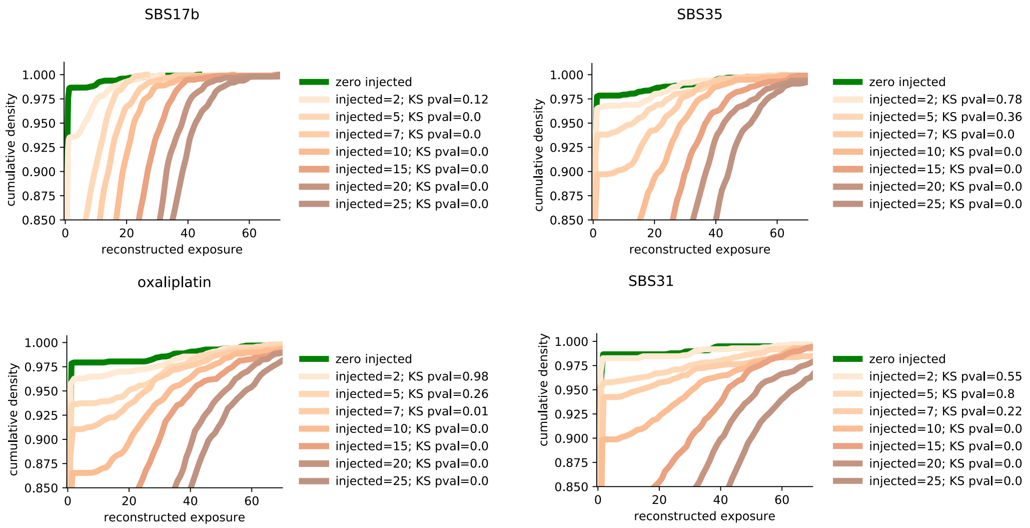

b

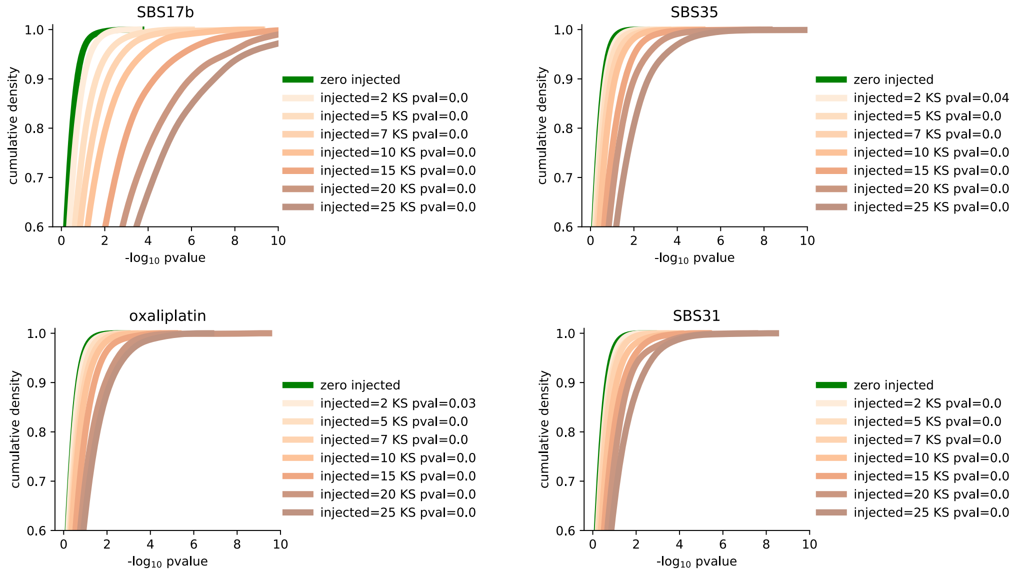

Supplement: Supplementary file 1 — Supplementary Information [file 41467_2021_24858_MOESM1_ESM.pdf]
