# Supplementary material for: The evolution of hematopoietic cells under cancer therapy
Source: Nat Commun. 2021 Aug 10;12:4803. doi: 10.1038/s41467-021-24858-3 (PMC8355079; doi:10.1038/s41467-021-24858-3)
Supplement: Supplementary file 3 — Reporting Summary [file 41467_2021_24858_MOESM3_ESM.pdf]

nature research

Corresponding author(s): Nuria Lopez-Bigas

Last updated by author(s): May 18, 2021

Reporting Summary

Nature Research wishes to improve the reproducibility of the work that we publish. This form provides structure for consistency and transparency in reporting. For further information on Nature Research policies, see our Editorial Policies and the Editorial Policy Checklist.

Statistics

For all statistical analyses, confirm that the following items are present in the figure legend, table legend, main text, or Methods section.

n/a	Confirmed	
☐	☒	The exact sample size (n) for each experimental group/condition, given as a discrete number and unit of measurement
☒	☐	A statement on whether measurements were taken from distinct samples or whether the same sample was measured repeatedly
☐	☒	The statistical test(s) used AND whether they are one- or two-sided<br>*Only common tests should be described solely by name; describe more complex techniques in the Methods section.*
☐	☒	A description of all covariates tested
☐	☒	A description of any assumptions or corrections, such as tests of normality and adjustment for multiple comparisons
☐	☒	A full description of the statistical parameters including central tendency (e.g. means) or other basic estimates (e.g. regression coefficient) AND variation (e.g. standard deviation) or associated estimates of uncertainty (e.g. confidence intervals)
☐	☒	For null hypothesis testing, the test statistic (e.g. F, t, r) with confidence intervals, effect sizes, degrees of freedom and P value noted<br>*Give P values as exact values whenever suitable.*
☒	☐	For Bayesian analysis, information on the choice of priors and Markov chain Monte Carlo settings
☒	☐	For hierarchical and complex designs, identification of the appropriate level for tests and full reporting of outcomes
☐	☒	Estimates of effect sizes (e.g. Cohen's d, Pearson's r), indicating how they were calculated

Our web collection on statistics for biologists contains articles on many of the points above.

Software and code

Policy information about availability of computer code

Data collection	-57 whole-genome sequenced AML samples from dbGAP phs000159 (informed consent of patients obtained from original study)<br>-2 samples of tAML from platinum-treated patients obtained from EGAD00001005028 (informed consent of patients obtained from original study)<br>-3 tAML samples sequenced in-house (Informed consent was obtained after approval by the Ethics Committee for Clinical Research of Hospital Santa Creu i Sant Pau; for their inclusion in this study approval was obtained by the Ethics Committee for Research of Hospital Clínic de Barcelona)<br>-261 paired-samples whole-exome sequenced AML cases obtained from GDC (informed consent of patients obtained from original study)<br>-347 whole-exome sequenced AML cases obtained from cbioPortal (informed consent of patients obtained from original study)<br>-Whole-genome mutations of metastases from 729 Breast, 537 Colon-Rectum, 154 Urinary-Tract and 155 Ovary primary tumors, retrieved from the Hartwig Medical Foundation (HMF) (DR-110) (informed consent of patients obtained from original study)<br>-Whole-genome somatic variants of 23 healthy blood samples were obtained from Osorio et al (ref 34) (informed consent of patients obtained from original study)
Data analysis	A) Mutation calling<br>The cram files were reverted to fastqs using bamtofastq. Then, the fastq files from phs000159, EGAD00001005028 and the inhouse cohort were processed in a uniform manner using the sarek pipeline implemented within nextflow-core (nextflow version 19.10). Briefly, the pipeline aligns the fastqs to GRCh38 using bwa-mem (0.7.17), and then implements GATK best practices to mark duplicates and base recalibration, and lastly somatic variant calling. Variant calling of both single nucleotide variants and short insertions and deletions was performed using Strelka2 (2.9.10). Only variants labeled as PASS by the algorithm were kept. Variants within regions of low mappability or low complexity were excluded from downstream analyses. All called somatic mutations were annotated with VEP (version 92).<br>B) Signatures extraction and analysis<br>We employed the SigProfilerJulia (bitbucket.org/bbglab/sigprofilerjulia) implementation carried out in our lab of the algorithm developed by Alexandrov et al (refs 5,63). Other analyses, such as the search for the platinum-associated signature in blood samples was done using the mSigAct algorithm.

C) Driver identification
We used the IntOGen pipeline
D) Clonal and subclonal mutations
We used the MutationTime.R package
E) Logistic regressions
We used multivariate logistic regression analysis

For manuscripts utilizing custom algorithms or software that are central to the research but not yet described in published literature, software must be made available to editors and reviewers. We strongly encourage code deposition in a community repository (e.g. GitHub). See the Nature Research guidelines for submitting code & software for further information.

Data

Policy information about availability of data

All manuscripts must include a data availability statement. This statement should provide the following information, where applicable:

- Accession codes, unique identifiers, or web links for publicly available datasets
- A list of figures that have associated raw data
- A description of any restrictions on data availability

The data employed in the paper is available through different sources. Whole genome sequences of samples in the WGS AML cohort are accessible through the dbGAP phs000159 (www.ncbi.nlm.nih.gov/projects/gap/cgi-bin/study.cgi?study_id=phs000159.v8.p4), EGAD00001005028 (ega-archive.org/datasets/EGAD00001005028) and EGAS00001005234 (in-house sequenced samples; ega-archive.org/datasets/EGAS00001005234) collections. Somatic mutations of samples in the WES AML cohort are available through the GDC repository provided by the authors in the original publication of the beat AML cohort (https://pubmed.ncbi.nlm.nih.gov/30333627/) and through the cbioportal (www.cbioportal.org). Whole genome sequences of tumor and blood samples in the metastasis cohort are available from the Hartwig Medical Foundation for academic research upon request (https://www.hartwigmedicalfoundation.nl/en). Blood somatic mutations identified in this cohort are also available from the Hartwig Medical Foundation for academic research upon request (https://www.hartwigmedicalfoundation.nl/en), due to the extreme difficulty to fully anonymize the data. A detailed description of this dataset of blood somatic mutations appears at www.biorxiv.org/content/10.1101/2020.10.22.350140.

Field-specific reporting

Please select the one below that is the best fit for your research. If you are not sure, read the appropriate sections before making your selection.

☒ Life sciences ☐ Behavioural & social sciences ☐ Ecological, evolutionary & environmental sciences

For a reference copy of the document with all sections, see nature.com/documents/nr-reporting-summary-flat.pdf

Life sciences study design

All studies must disclose on these points even when the disclosure is negative.

Sample size	-30 WGS tAML samples<br>-32 WGS primary AML samples<br>-40 WGS platinum-exposed breast cancer metastatic samples<br>-78 WGS platinum-exposed ovarian cancer metastatic samples<br>-46 WGS platinum-exposed urinary tract cancer metastatic samples<br>-198 WGS platinum-exposed colorectal cancer metastatic samples<br>-208 WGS 5FU-exposed colorectal cancer metastatic samples<br>-126 WGS 5FU-exposed breast cancer metastatic samples<br>-23 WGS healthy blood samples<br>-608 WES primary and tAML samples<br>-3,785 WGS non-malignant blood samples from metastatic cancer patients
Data exclusions	All samples in the aforementioned datasets were included in the analyses.
Replication	Analyses were carried out in the aforementioned datasets.
Randomization	In the analysis of mutational signatures in clonal hematopoiesis, we generated synthetic mutational catalogues with/without mutations contributed by a foreign signature to ascertain the presence/absence of the signature.
Blinding	The analyses carried out in the study required no blinding.

Reporting for specific materials, systems and methods

We require information from authors about some types of materials, experimental systems and methods used in many studies. Here, indicate whether each material, system or method listed is relevant to your study. If you are not sure if a list item applies to your research, read the appropriate section before selecting a response.

Materials & experimental systems

n/a	Involved in the study	
☒	☐	Antibodies
☒	☐	Eukaryotic cell lines
☒	☐	Palaeontology and archaeology
☒	☐	Animals and other organisms
☐	☒	Human research participants
☒	☐	Clinical data
☒	☐	Dual use research of concern

Methods

n/a	Involved in the study	
☒	☐	ChIP-seq
☒	☐	Flow cytometry
☒	☐	MRI-based neuroimaging

Human research participants

Policy information about studies involving human research participants

Population characteristics	Three patients with prior history of a solid malignancy suffering from a secondary AML.
Recruitment	The three patients were recruited by the hematology department of Hospital Sant Pau.
Ethics oversight	Informed consent was obtained from three secondary AML patients after approval by the Ethics Committee for Clinical Research of Hospital Santa Creu i Sant Pau. Within the context of the tAML study described in this paper, the use of these samples was approved by the Ethics Committee for Research of Hospital Clínic de Barcelona.

Note that full information on the approval of the study protocol must also be provided in the manuscript.
